# In Vitro and Computational Response of Differential Catalysis by *Phlebia brevispora* BAFC 633 Laccase in Interaction with 2,4-D and Chlorpyrifos

**DOI:** 10.3390/ijms252312527

**Published:** 2024-11-22

**Authors:** Alan Rolando Ayala Schimpf, Laura Ester Ortellado, Marcelo Daniel Gamarra, María Isabel Fonseca, Pedro Darío Zapata

**Affiliations:** 1 Laboratorio de Biotecnología Molecular, Instituto de Biotecnología de Misiones “Dra. Maria Ebe Reca” (InBioMis), Facultad de Ciencias Exactas Químicas y Naturales, Universidad Nacional de Misiones, Posadas 3300, Misiones, Argentina; ortelladolauraes@gmail.com (L.E.O.); mdgamarraok@gmail.com (M.D.G.); fonsecamariaisabel@yahoo.com.ar (M.I.F.); pedro.zapata@unam.edu.ar (P.D.Z.); 2 Consejo Nacional de Investigaciones Científicas y Técnicas (CONICET), Buenos Aires C1425FQB, Argentina

**Keywords:** white rot fungi, laccase, pesticides, enzymatic activity, molecular dynamics

## Abstract

Enzymes secreted by white rot fungi (WRF), such as laccase, offer a promising approach for the treatment of hazardous xenobiotic compounds. This study conducted a comprehensive analysis of the impact of the pesticides 2,4-dichlorophenoxyacetic acid (2,4-D) and chlorpyrifos on the laccase of *Phlebia brevispora* BAFC 633 through in vitro and bioinformatics analyses. The fungal strain was shown to be tolerant to both pesticides, with notable morphological and ultrastructural alterations in the mycelium. Laccase activity and two isoenzymes (53 and 70 kDa) were detected in all initial treatments. The laccase was concentrated for subsequent catalytic evaluation in the presence of both pesticides, showing high stability at a pH of 3.6 and a temperature range of 50–60 °C. The *lacI* gene, corresponding to this laccase, was modeled, and its structure revealed a defined catalytic pocket validated with a drug score of 0.61. Molecular docking estimated affinity energies of −5.06 and −9.41 Kcal mol^−1^ for 2,4-D and chlorpyrifos, respectively. Molecular Mechanics Poisson–Boltzmann Surface Area (MM/PBSA) analysis through 250 ns of molecular dynamics revealed stronger hydrophobic interactions of laccase with chlorpyrifos and highlighted the importance of residue His460 in stabilizing both complexes. Understanding the impact of these agrochemicals on the catalytic function of laccase is crucial for developing future biotechnological strategies involving this enzyme.

## 1. Introduction

Agrochemicals are used in agriculture to control pests and phytopathogens, but their excessive use has raised serious environmental concerns [1]. Two of the most active pesticides on a global scale are 2,4-dichlorophenoxyacetic acid (2,4-D) and chlorpyrifos. The former is a herbicide widely used to control broadleaf weeds in crops such as corn and small grains [2]. Chlorpyrifos, on the other hand, is an organophosphate pesticide commonly used in agriculture as an insecticide and as a biocide to control pests [3]. The toxicological profile of 2,4-D indicates that it is highly mobile in soil and has the potential to migrate to groundwater, which is of particular concern for households near areas of high application [4]. Chlorpyrifos residues have also been found in soils, natural waters, food, animals, and humans [3].

*Phlebia brevispora* BAFC 633 is a WRF that has shown potential in biotechnological applications for the degradation of contaminating compounds, as well as in the biopulping of wood chips [5,6]. In addition, it has been studied for its potential in the bioremediation of lignocellulosic waste and the production of value-added products [7]. It is characterized by its metabolic capacity to mineralize polymeric complexes, which is attributed to the secretion of an enzymatic complex among which laccase (EC 1.10.3.2, *p*-diphenol: dioxygen oxidoreductase) plays a major role [8]. Laccases belong to the family of the multicopper oxidases and catalyze the oxidation of a wide range of phenolic compounds and aromatic or aliphatic amines by transferring four electrons to their catalytic center with the consequent reduction of molecular oxygen to water [8]. Structurally, fungal laccases have three domains and four Cu atoms distributed across the type 1 (T1), type 2 (T2), and binuclear type 3 (T3) sites, which are differentiated according to their spectroscopic characteristics. The mononuclear T1 center, blue due to a maximum absorbance of around 600 nm, is where the oxidation of the substrates occurs in the presumed active positions through a histidine H bond [9]. *P. brevispora* BAFC 633 produces laccases in high proportions, which are effective in the degradation of complex organic compounds [10,11]. Recently, the laccases expressed in *Pichia pastoris* were used for the degradation of synthetic dyes [7,10]. The utility of the *P. brevispora* BAFC 633 laccase immobilized on nanoporous aluminum oxide has also been proven for the treatment of black liquor, demonstrating superior stability and reuse efficiency compared to the free enzyme, thereby suggesting a great potential for its application under industrial conditions [12]. The success of using candidate enzymes to treat contaminants such as pesticides depends on the detailed knowledge of the biological catalyst’s response to hostile environments. Evaluating this response is crucial for the practical and economical applications of these enzymes in environmental sanitation. Bioinformatics studies and in vitro research are required to fully understand the mechanisms of pesticide degradation and to develop strategies for their bioremediation [13]. However, no bioinformatics laccase affinity studies on xenobiotic compounds relevant to environmental biotechnology have been reported in *P. brevispora* BAFC 633, the importance of which is described in recent studies [11,14,15].

Proteins are biologically dynamic molecules, and their flexibility properties are vital for determining their molecular mechanisms and ligand recognition; in this sense, the use of chemoinformatics tools based on molecular docking simulations is an effective strategy for predicting laccase enzyme interactions with various substrates [16]. Among them, molecular dynamics (MDs) is an optimal computational method for obtaining structural and dynamic information about proteins, which is important for understanding their stability and flexibility in interactions with other molecules [17,18].

The objective of this study was to perform a comprehensive analysis of laccase activity, assessing its stability under both in vitro and in silico conditions, in response to exposure to the pesticides 2,4-D and chlorpyrifos. This will allow for an in-depth understanding of how these substances impact their catalytic function in *P. brevispora* BAFC 633.

## 2. Results and Discussion

### 2.1. Effect of Pesticides on Growth and Laccase Secretion

The tolerance of WRF to xenobiotics varies according to the structure and concentration of the compounds, as well as nutritional and environmental conditions [19]. Recent studies have demonstrated the potential of fungi belonging to the genus *Phlebia* sp. for the treatment of chlorpyrifos-contaminated soils [20]. However, no studies of this genus against 2,4-D have been reported.

**Figure 1:**
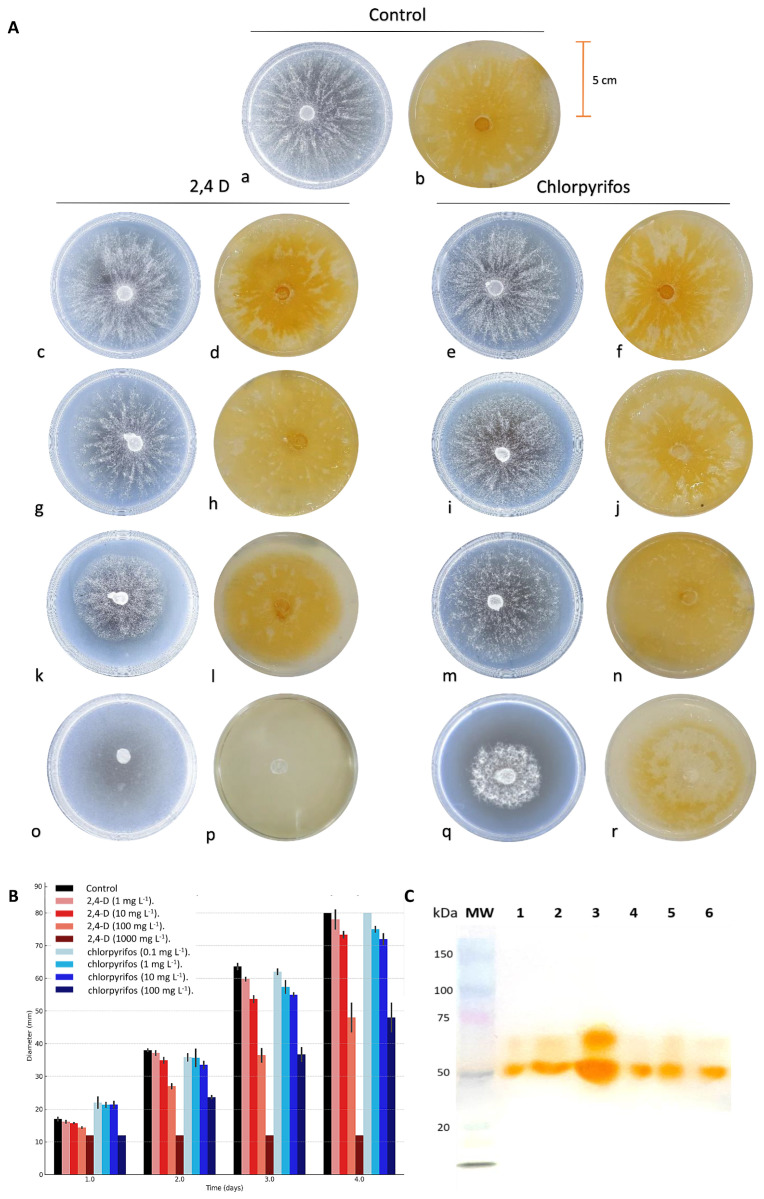
Growth pattern of *P. brevispora* BAFC 633 and detection of laccase activity in solid media supplemented with different concentrations of 2,4-dichlorophenoxyacetic acid (2,4-D) and chlorpyrifos (CP) (**A**). Macroscopic view (frontal) of mycelial appearance on solid medium containing 12.7 g L^−1^ malt extract and 20 g L^−1^ agar (MEA) (**left**) and laccase secretion on MEA with 2,6-dimethoxyphenol (DMP) (**right**), in the absence of pesticides (**a**,**b**); with 2,4-D at [1 mg L^−1^] (**c**,**d**), [10 mg L^−1^] (**g**,**h**), [100 mg L^−1^] (**k**,**l**), and [1000 mg L^−1^] (**o**,**p**); and with chlorpyrifos at [0.1 mg L^−1^] (**e**,**f**), [1 mg L^−1^] (**i**,**j**), [10 mg L^−1^] (**m**,**n**), and [100 mg L^−1^] (**q**,**r**). (**B**) Modeling fungal growth of *P. brevispora* BAFC 633 in solid medium. (**C**) Enzymatic profile obtained through SDS-PAGE and incubated with DMP for *P. brevispora* BAFC 633. (1) 2,4-D [1 mg L^−1^]. (2) 2,4-D [100 mg L^−1^]. (3) *P. brevispora* BAFC 633 without pesticides. (4) CP [100 mg L^−1^]. (5) CP [1 mg L^−1^]. (6) CP [0.1 mg L^−1^]. (MW): molecular weight.

Using predictive mycology, the degree of inhibition of 2,4-D and chlorpyrifos on the fungal strain was determined by evaluating the parameters k and τ. The average values of k and τ were 1.30 ± 0.02 and 2.13 ± 0.03, respectively, for the control sample, while for 2,4-D they varied between 1.21 ± 0.08 and 0.58 ± 0.10 for k, and between 2.22 ± 0.02 and 3.54 ± 0.30 for τ; for chlorpyrifos, the values ranged between 1.15 ± 0.06 and 0.66 ± 0.10 for k, and between 2.16 ± 0.07 and 3.55 ± 0.27 for τ (*p* < 0.05) (Table 1). In the present study, *P. brevispora* BAFC 633 was able to grow in the presence of all chlorpyrifos concentrations while with 2,4-D it grew up to the assay at 100 mg L^−1^ (Figure 1A: left).

Pesticide tolerance is often assessed by macroscopic effects, excluding possible unseen cellular consequences [21]. The tolerance assessment of *P. brevispora* BAFC 633 to both pesticides is summarized by Δτ values (Table S1).

The fungus grew rapidly in the control medium without pesticides (τ = 4.3 days) and decreased successively at higher concentrations of both pesticides (Figure 1A left,1B and Table 1). It is important to note that the strain had similar growth behavior in the presence of both pesticides at 10 mg L^−1^, where the growth curves are almost superimposed (Figure 1B). The ability of fungi to grow in the presence of contaminating compounds is crucial for their application in bioremediation [22,23]. Although some contaminants can negatively affect the growth rate, this does not invalidate their use [24]. The predictive mycology method demonstrated the growth inhibition of *P. brevispora* BAFC 633 by 2,4-D, similar to that observed in Basidiomycota fungi exposed to atrazine, another widely used herbicide [25].

All treatments, including controls with and without pesticides, showed laccase activity (Figure 1A: right). The production of this enzyme is essential for the adaptation and survival of fungi in contaminated environments [26] and can remediate various recalcitrant compounds such as chlorpyrifos [27]. The diameter and color intensity of the halos formed by DMP oxidation were dependent on the mycelial growth present at each pesticide concentration. The enzymatic activity expanded radially from the edge of the colony and was less intense at high pesticide concentrations. A similar color intensity was observed in the halos corresponding to the chlorpyrifos and 2,4-D treatments.

The molecular mass of fungal laccases ranges between 50 and 100 kDa [28], and up to 30% of their molecular weight may be composed of carbohydrates [29,30]. An SDS-PAGE analysis with DMP showed the presence of a 53 kDa isoenzyme and a 70 kDa isoenzyme in all conditions analyzed (Figure 1C). The 53 kDa isoenzyme detected is consistent with that reported by Fonseca (2010) [31], for the same copper-induced *P. brevispora* BAFC 633 strain. Other fungi such as *Pleutotus pulmonarius* LBM 105 have also shown a 53 kDa laccase in the presence of contaminants such as polychlorinated biphenyls (PCBs) after 21 days of culture [32], in addition to a 35 kDa laccase induced by the presence of citrus industry effluents in a solid medium [33].

This would explain the appearance of different laccase isoforms depending on the incubation period, the culture medium used, and the toxicity of the substrates. Although laccase isoenzymes induced by the presence of chlorpyrifos have not been previously reported, recent studies have documented its degradation by laccase [20], suggesting that the toxicity of chlorpyrifos could also be linked to the expression of specific isoenzymes.

### 2.2. Effect of Pesticides on Fungal Morphology

**Figure 2:**
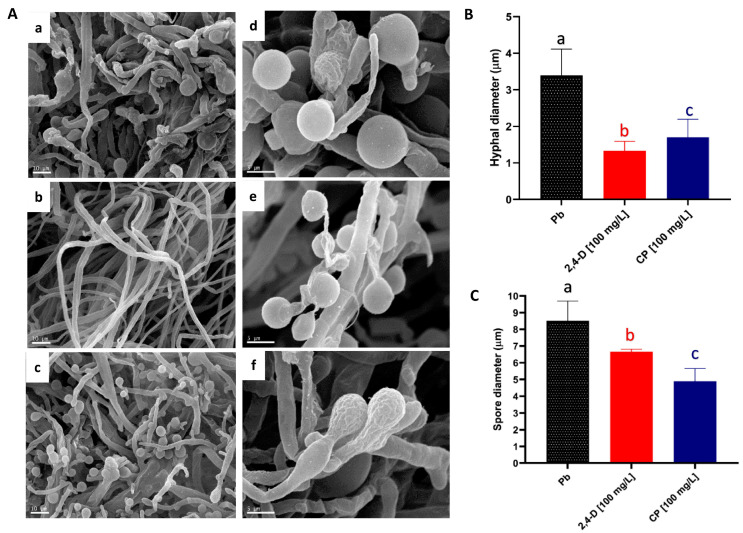
Ultrastructural analysis of *P. brevispora* BAFC 633 in the presence of pesticides. (**A**) Scanning electron micrographs of *P. brevispora* BAFC 633 grown on MEA medium: (**a**) hyphae in control conditions without pesticides, (**b**) hyphae in the presence of 2,4-dichlorophenoxyacetic acid (2,4-D) [1000 mg L^−1^], (**c**) hyphae in the presence of chlorpyrifos (CP) [10 mg L^−1^]; (**d**) basidiospores in control conditions without pesticides, (**e**) basidiospores in the presence of 2,4-D [1000 mg L^−1^], and (**f**) basidiospores in the presence of chlorpyrifos [10 mg L^−1^]. (**B**) Comparison of mean diameters of hyphae and spores (**C**) of *P. brevispora* BAFC 633 with 12 days of incubation in medium with 2,4-D, CP, and their control (without pesticides). Mean diameters with different letters indicate a significant difference (*p* < 0.05). For each image obtained via SEM, 50 measurements were taken using the ImageJ 1.46r (National Institutes of Health (NIH) Bethesdam MD, EE.UU) program, and statistical analysis was performed with Statgraphics Centurion XVI.I (Statgraphics Technologies, The Plains, VA, USA).

Exposure to xenobiotics can alter the morphology and physiology of microorganisms, causing the collapse of fungal structures such as hyphae and spores [34]. In this study, morphological changes were observed at the macroscopic level in *P. brevispora* BAFC 633 exposed to both pesticides, resulting in a more compact mycelium with reduced hyphal development (Figure 1A: left). Under exposure to 2,4-D, mycelial development showed morphological modifications with respect to the control. These changes in the mycelium, which were observed from a contaminant concentration of 100 mg L^−1^, were also observed under exposure to chlorpyrifos but from a concentration of 10 mg L^−1^. Studies with *Aspergillus flavus Lynk* exposed to penconazole or cypermethrin showed changes in its hyphae, such as terminal blisters and vesicles, as well as shrinkage, deformation, and swelling [35]. These morphological transformations are usually correlated with herbicide concentration [36]. Observational analysis at the ultrastructural level revealed that *P. brevispora* BAFC 633 hyphae exposed to a pesticide medium had a significantly smaller diameter than the control hyphae (*p* < 0.05) (Figure 2).

The branching density and mycelial network are influenced by environmental and nutritional factors [37]. Although suggesting possible changes in the hyphal length, the arrangement in the photomicrograph did not allow for confirmation. Differences were also observed in the spore diameter between the control and media with both pesticides (*p* > 0.05), with more pronounced effects noted in the chlorpyrifos treatment (Figure 2C). These morphological adaptations represent fungal strategies to cope with toxic conditions, enabling growth toward less adverse areas with minimal resource consumption [38,39].

### 2.3. Pesticide-Induced Changes in Laccase Activity and Stability

**Figure 3:**
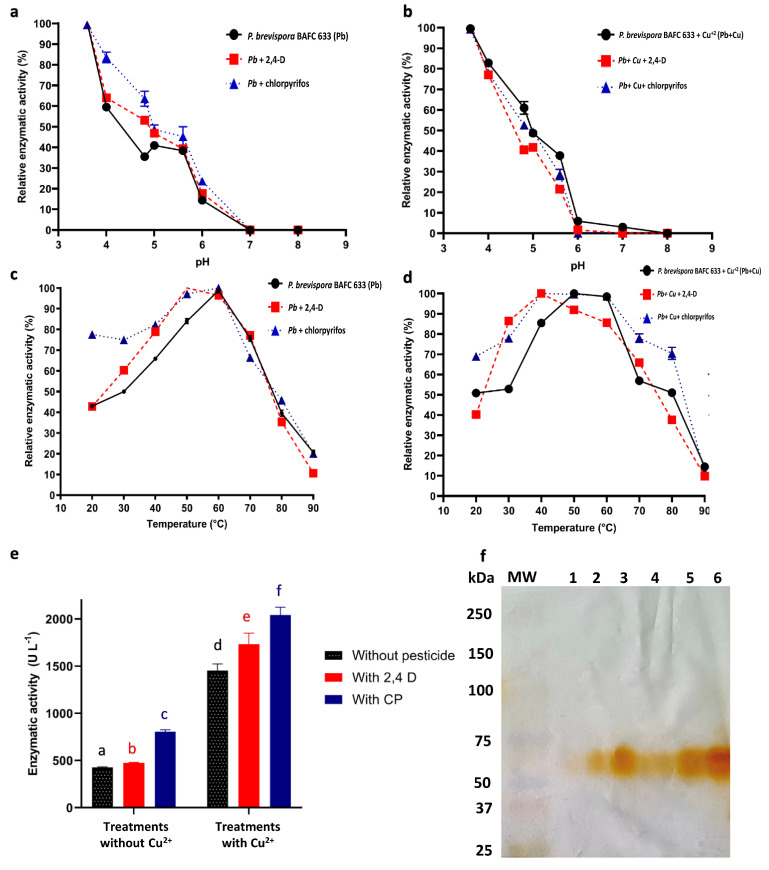
Effect of pesticides on laccase activity. (**a**–**d**) Effect of pH and temperature on laccase activity in the concentrated enzyme fraction of *P. brevispora* BAFC 633, after its production in the absence (left) and presence (right) of Cu^2+^ [1 mM] (●), and its effect in the presence of 2,4-D (■) and chlorpyrifos (CP) (▲). Data are expressed as relative enzyme activity (%), with error bars representing standard deviations of triplicate measurements. Statistical analysis (ANOVA followed by Tukey’s post hoc test) revealed no significant differences (*p* > 0.05) between treatments at different pH and temperature conditions. (**e**) Effect of pesticides on laccase activity achieved under optimal pH and temperature conditions. Data are represented as means ± standard deviations (SDs) of three replicates. Means with different letters are significantly different from each other (*p* < 0.05) according to a *t*-test. (**f**) Enzyme profile obtained through SDS-PAGE and incubated with DMP for the purified laccase fraction of *P. brevispora* BAFC 633. Lanes 1–6 represent laccase fractions from different independent inoculations.

The effects of chlorpyrifos and 2,4-D on laccase enzyme activity in WRF have not been previously studied, highlighting the little attention paid to the latter compound. In this study, the production of laccase was carried out with and without copper sulfate, which is recognized as an inducer of this enzyme for *P. brevispora* BAFC 633 [21], as well as for other WRF [40,41,42,43]. The concentrated fractions of this enzyme were verified through SDS-PAGE (Figure 3f), where only the presence of the 53 kDa isoenzyme was observed.

The optimal pH value for all treatments was 3.6 (Figure 3a,b). These results were also consistent with those described in the literature for other fungal laccases with optimal activities at an acidic pH [10,33,44,45,46,47,48,49,50]. In these studies, enzymatic activity sharply decreased to an almost undetectable level as the pH approached neutral, possibly due to the binding of the hydroxide ion to the T2/T3 site of the enzyme [51]. Under the previously determined conditions, the optimum temperature recorded was 60 °C both in the presence and absence of chlorpyrifos, while it was 50 °C with 2,4-D (Figure 3c). The maximum activity in the presence of both pesticides, using the laccases produced in the presence of copper (Cu^2+^), was recorded at 40 °C, while in the absence of contaminants it was 50 °C (Figure 3d). The optimum temperature of the laccase (50–60 °C) remained constant in the presence of both pesticides, in line with previous studies of WRF laccases exposed to inducers and contaminating substrates [33,52]. A maximum activity between 25 and 80 °C has been reported for laccases [53], with most having optimal values between 50 and 60 °C. This variation in the optimal temperature may be due to conformational changes and the stabilization of the active site dependent on the laccase–pesticide complex formed. These changes result in a more or less favorable conformation for substrate binding and catalysis, which can influence the temperature at which the enzyme exhibits optimal activity [54,55].

Enzyme stability is a desired characteristic in commercial-scale enzyme production and represents an important property for any industrial application [56]. The laccase enzyme exhibited high pH stability with both pesticides in the absence of Cu^2+^, maintaining over 4 h of half-life within the pH range of 4.8 to 7. At pH 3.6, 50% of its activity remained after approximately 4 h. In the presence of Cu^2+^, more than 70% of enzymatic activity was retained after 4 h with both pesticides (Figure S1b; Table S2). In addition to its relevance for industrial applications, the stability of laccase across different pH and temperature ranges is crucial for bioremediation, where contaminated environments can present variable and challenging conditions. Polluted soils and water bodies often experience pH and temperature fluctuations that may impact enzymatic activity. Therefore, the ability of laccase to maintain its activity over a wide range of conditions is essential for contaminant degradation in real, uncontrolled settings. This reinforces its potential as a tool for xenobiotic remediation, where adaptability to environmental pH and temperature changes is a critical requirement.

Regarding temperature, in the presence of 2,4-D, up to 100% of laccase activity was retained after 2 h of incubation at 40 °C and more than 80% of its activity was retained at 30 and 70 °C, with activity being drastically reduced at the latter (Figure S1). Furthermore, in the presence of Cu^2+^, the half-life of laccase increased at 20, 30, 40, and 50 °C, with no significant variations at 60 and 70 °C. In an incubation with chlorpyrifos for 2 h, the enzyme retained more than 50% of its activity in all treatments and more than 70% at 20, 40, and 60 °C; in the presence of Cu^2+^, the half-life of the enzyme increased in all treatments at different temperatures (Table S2). In general terms, laccases have been reported to remain stable at between 50 and 70 °C [46,56]. In this study, laccase maintained its half-life for more than 5 h at temperatures between 20 and 40 °C. The thermostability of laccase showed similar responses to both pesticides (slightly higher with 2,4-D), in agreement with the results reported by Fonseca (2015) [10], as the same enzyme in the absence of pesticides, suggesting that they do not substantially modify its biochemical properties.

The effect of chlorpyrifos and 2,4-D on laccase activity was studied under the optimal activity conditions mentioned above. The enzymatic activity in the presence of 2,4-D (474.19 U L^−1^) remained similar to the control without pesticides (425.13 U L^−1^); however, in contact with chlorpyrifos, a significant increase was recorded, reaching 805.26 U L^−1^. The above suggests that chlorpyrifos may act as an inducer or stabilizer of the laccase enzyme, which, bound to the catalytic site, stabilizes the enzyme–substrate conformation more effectively than 2,4-D. In addition, the chemical structure of chlorpyrifos, which differs from that of 2,4-D, could interact more favorably with the amino acids of the laccase active site, increasing its catalytic activity [54,55]. In the treatments of *P. brevispora* BAFC 633 grown in a culture medium with Cu^2+^, a laccase activity of 1451.92 U L^−1^ was recorded, with a significant increase in the presence of 2,4-D and chlorpyrifos, with values obtained of 1730.46 U L^−1^ and 2040.33 U L^−1^, respectively (Figure 3e).

The results of this study indicate that the pesticides 2,4-D and chlorpyrifos significantly affect the laccase activity of *P. brevispora* BAFC 633. This effect may be due to their ability to interact directly with the enzyme structure, causing changes in its three-dimensional conformation that enhance its catalytic activity. Such an interaction can stabilize the active form of laccase or facilitate the binding of substrates, which ultimately improves its efficiency. Furthermore, pesticides can influence the microscopic environment surrounding the enzyme, modifying solvent properties or the substrate’s accessibility to the active site. These environmental adjustments promoted by pesticides may create more optimal conditions for catalysis, which would partly explain the observed increase in enzymatic activity.

### 2.4. Model Generation and Structural Validation

Various structures of bacterial and fungal laccases have been determined using crystallography [57]. In homologous modeling, the selection of a suitable template is crucial. *LacI* was modeled using the crystallographic structure of *T. versicolor* (PDBID: 1GYC) as a template with 65% identity and 100% confidence values (Figure S2). According to Pearson (2013) [58], sequences with more than 40% identity are considered to be functionally similar. For *lacI*, the identity was greater than 60%, ensuring an accurate model that exceeded the minimum requirement [59]. In addition, it exhibited the typical molecular architecture of laccases, with three clearly identified cupredoxin domains [57]. The copper ions were colocalized with their correct coordination distances (Table S3). The MolProbity evaluation yielded a score of 1.33 and a QMEAN value of 0.82. The validation of the 3D model was essential to evaluate stereochemical parameters and folding accuracy.

The Ramachandran diagram prepared using UCSF Chimera showed that 86.2% of the residues in the generated structure occupied permitted regions. The residues located in non-allowed regions were of the order of 1%, corresponding mostly to glycine (G) and proline (P). In general, the architectures of the models were in agreement with previously reported laccases [40]. Overlap analysis showed that *lacI* was similar to the 1GYC template, showing few regions with significant differences (greater than 3 Å) (Figure S2). The analysis suggests that the concatenated structures exhibited negligible overall divergence with a deviation of 1.03 Å between them and a root mean square deviation (RMSD) of 1.23 Å for all atoms in specific regions from the templated sequence. VADAR-estimated weights and accessible surface area values demonstrated enzymatic features, such as cavities and pockets in various regions of the proteins [41]. The detailed report of the VADAR analysis is summarized in Table S4.

### 2.5. Characterization of Ligand Binding Pockets

**Figure 4:**
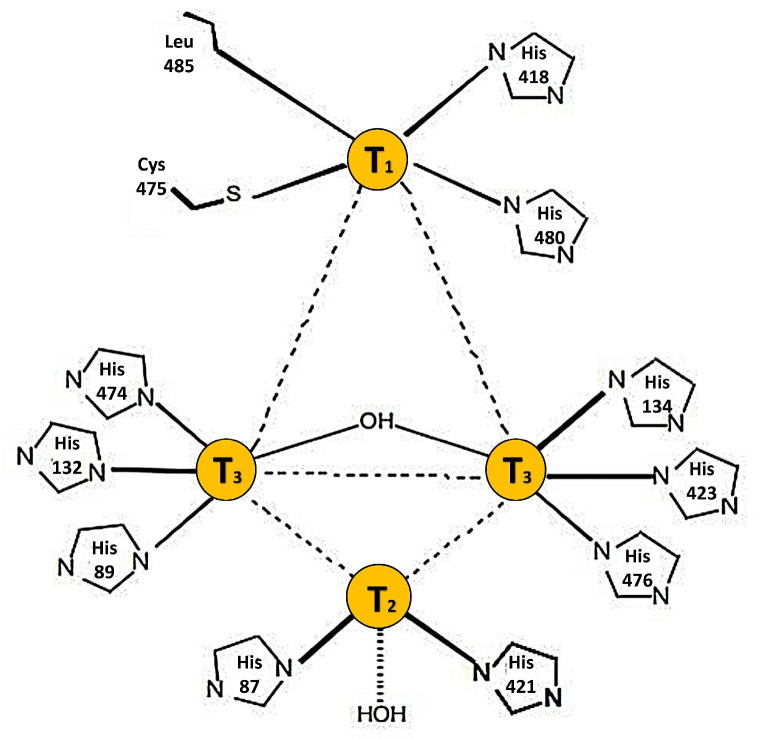
Structural representation of the catalytic site of laccase *lacI*, showing the key amino acids involved in copper ion coordination and ligand interaction at the T1 site.

The active site of an enzyme provides an optimal catalytic environment for the binding of chemical compounds [42] (Figure 4).

**Figure 5:**
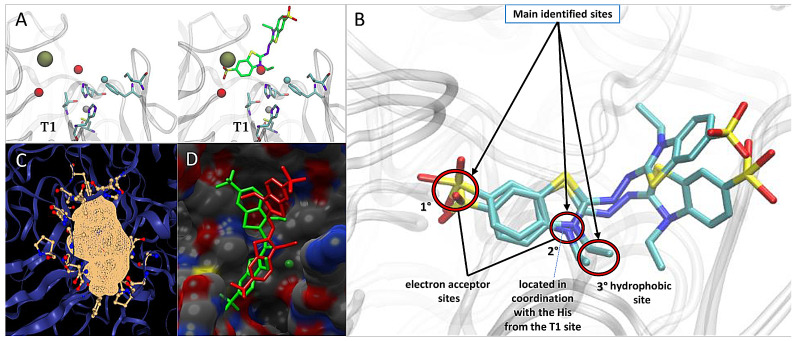
Structural analysis of the catalytic site T1 in *lacI* from *P. brevispora* BAFC 633. (**A**) Ideal interactions calculated at the T1 site (**left**). Sites aligned with the ABTS crystal of the laccase structure of the bacterium *Bacillus subtilis* encode PDB:3ZDW (**right**). The red spheres are electron acceptor sites, and the brown spheres are aromatic sites. (**B**) Main ligand interaction sites identified in *lacI* of *P. brevispora* BAFC 633. (**C**) Surface of the T1 catalytic pocket; images were obtained from DoGSiteScorer. (**D**) Comparison between the best poses with the co-crystallized 3ZDW structure. The ligand in the docking complex is depicted in green and 3ZDW in red. Images were obtained from Chimera 1.14.

DoGSiteScorer estimated a total of 62 pockets on the surface of *lacI*. Taking into account the druggability as well as the location of the pockets in the protein structure, the pocket in the position coinciding with the active site T1 was selected (Figure 5C). The pocket selected by DoGSiteScorer consisted of 18 different amino acids (Asp173, Tyr175, Pro185, His186, Leu187, Ala188, Asp228, Pro229, Ser230, Phe261, Ala262, Pro285, Asn286, Ala287, Gln288, Gly415, Gly416, and Pro417). This pocket had a surface area of 383.22 Å^2^ and a drug score of 0.61. Drug score values between 0.5 and 1.0 indicate a druggable pocket, with values close to 1.0 being the most desirable [43]. The group of pocket-forming residues was predominantly hydrophobic.

When the structures were compared with ABTS (PDB ID: 3ZDW) and the *lacI*-ABTS docking complex, a divergence in atomic distances was observed, resulting in distinct patterns of laccase surface topology. This could be attributed to steric hindrances caused by certain residues in the models, which make it difficult to adopt configurations similar to those of the 3ZDW complex. Previous studies have shown that the binding behavior of ABTS in laccases such as those from *T. versicolor* depends on the pH and the protein’s oxidation state, factors that also influence the orientation and accessibility of key residues at the binding site [60].

The calculation of ideal sites to identify potential biases in the active site of the generated model revealed a major electron acceptor site interacting with one of the histidine (H)-coordinating copper atoms (Figure 5A). In addition, another acceptor site was observed, showing a polar environment capable of stabilizing the binding of polar groups such as SO in ABTS. Finally, an aromatic site was identified close to the pocket entrance. A validation of the docking using these biases in the laccase model was compared with the 3ZDW crystal with co-crystallized ABTS (Figure 5B,D). In the docking simulations performed, no output was obtained below the 2 Å cutoff point. However, the RMSD values closest to the structure of *Bacillus subtilis* with co-crystallized ABTS were selected. The docking result for *lacI* selected was 5540 Å for the RMSD value with a binding energy of −9.41 Kcal mol^−1^. The resulting interaction confirmed the active site residues in the *lacI* receptor.

### 2.6. Molecular Docking Analysis

**Figure 6:**
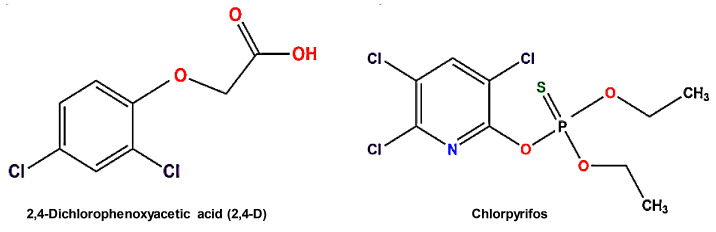
Structural representation of chlorpyrifos and 2,4-d pesticides used in molecular docking analyses (red = oxygen, blue = nitrogen, green = sulfur).

Computational simulations have proven to be an invaluable tool for estimating the molecular factors that influence the interaction of a protein with a specific ligand. On the one hand, biased molecular docking methods are instrumental in predicting the most likely way in which a molecule can bind to its target protein, providing an objective assessment in terms of both energy and accuracy [61]. On the other hand, molecular dynamics (MDs) simulations are essential due to their ability to represent the conformational diversity of a protein [62] and to reveal how a protein–ligand complex behaves in solution, allowing for the assessment of its stability over time [63]. In this study, adaptations were made to the energy grids for the receptor laccase, taking into account previously identified pharmacophoric sites and catalytic site residues. These modifications are an essential component to improve the accuracy of the docking process [62]. Preliminary results indicated that the protocol was able to reproduce the ABTS binding mode, although some differences were observed due to the variations exhibited by *lacl* compared to the reference receptor (*B. subtilis* laccase with co-crystallized ABTS, PDB ID: 3ZDW), displaying an energetics value of −9.41. To analyze in detail the interaction patterns of the ligands, molecular docking analyses were performed with the pesticides of interest (Figure 6).

**Figure 7:**
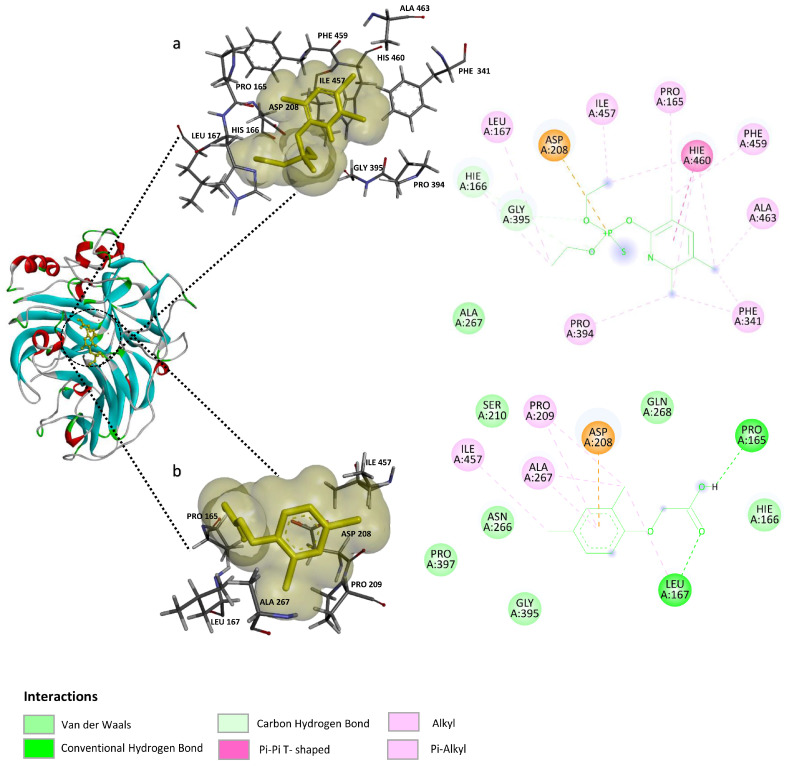
Interactions of *lacI* from *P. brevispora* BAFC 633 with chlorpyrifos (top) and 2,4-dichlorophenoxyacetic acid (2,4-D) (bottom). The interacting amino acid residues in each ligand–receptor complex are shown in 2D: (**a**) *lacI*–chlorpyrifos and (**b**) *lacI*-2,4-D. The 3D surface view represents the binding pocket in each complex.

Chlorpyrifos showed the best energetic results, with a binding energy of −7.44 Kcal mol^−1^, in which the main contributors to the interaction were aliphatic and aromatic amino acids, notably residues His166, His460, and Phe341 (Figure 7a). In contrast, 2,4-D showed an energy of −5.06 Kcal mol^−1^, indicating a weaker interaction due to the lack of Pi-stacking-type interactions observed in the complex with chlorpyrifos. The compound 2,4-D bound to a site contiguous to T1, showing coordination between its carboxyl group and the Asp208 residue as the main interaction (Figure 7b). Details of the residues involved in the interactions with both pesticides are presented in Table 2.

### 2.7. MDS: Qualitative Analysis of lacI Binding to Chlorpyrifos and 2,4-D

**Figure 8:**
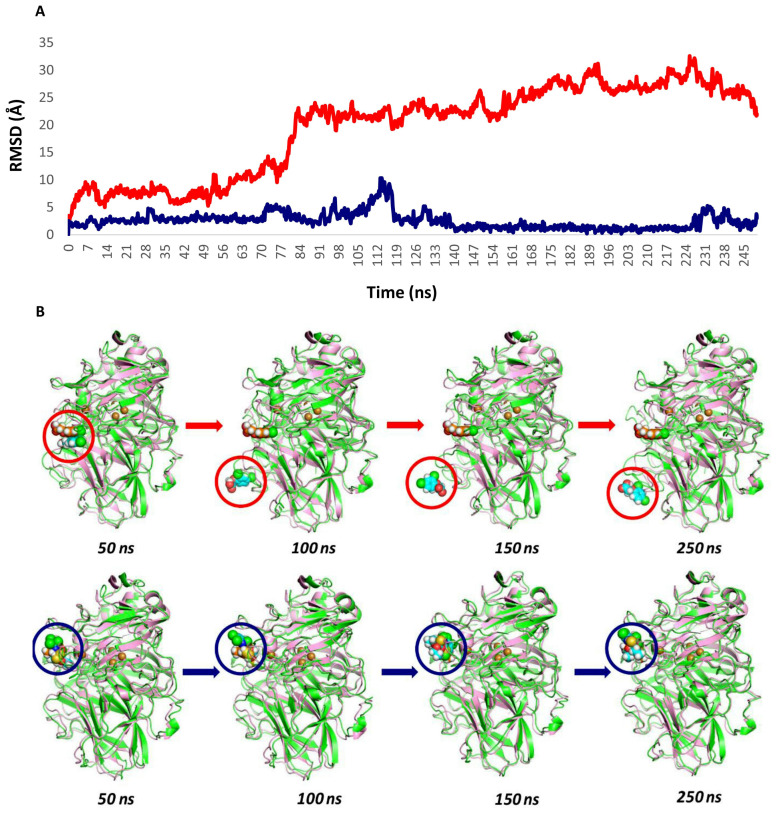
(**A**) Representation of the root mean square deviation (RMSD) during the DM simulation at 250 ns of *lacI* with 2,4-dichlorophenoxyacetic acid (2,4-D) (▬) and chlorpyrifos (CP) (▬). (**B**) Conformational transformation of complexes in the *lacI*-2,4-D system (top) and *lacI*-CP (bottom) during the MDS for 250 ns. The red and blue circles follow the position of each ligand on the laccase receptor during the trajectory.

In the case of *lacl*–chlorpyrifos, molecular dynamics simulations showed a steady trend of active site stability along the trajectories, with an RMSD that remained within the 2 Å range (Figure 8A). The main stabilization was attributed to the interaction of the benzene ring of chlorpyrifos with residues Phe341 and Asn462. Although some RMSD variations were observed outside this range, visual analysis revealed that the compound could move longitudinally in the same plane, while consistently maintaining its binding mode, mainly due to its interaction with Asn462. This likely explains the RMSD variations without a loss of ligand binding. This event was observed in all repetitions of the simulations performed, with stability maintained throughout the entire protein structure, reinforcing the validity of the model constructed.

On the other hand, the *lacl*-2,4-D complex showed markedly different dynamics, with the RMSD fluctuating in the 20–30 Å range. At the beginning of the simulation, there was a strong interaction with the Asp208 residue. However, throughout the trajectories, a change in the 2,4-D binding site was observed, moving to a position entirely opposite to the T1 site (Figure 8B). This fact may explain the high RMSD ranges and rule out binding loss due to instability. Table 3 summarizes the MDs trajectory analysis for both substrates.

It is important to note that, although the RMSD of the *lacI*-2,4-D complex fluctuated within the 20–30 Å range, a more detailed analysis of the molecular dynamics simulation revealed that 2,4-D exited the catalytic pocket at around 80 ns. This observation, which was only made possible by the extended 250 ns simulation, highlights the transient nature of the interaction between 2,4-D and the catalytic site. The prolonged simulation was crucial for capturing this event, underscoring the importance of conducting longer MDs simulations to fully understand ligand behavior within the catalytic environment.

**Figure 9:**
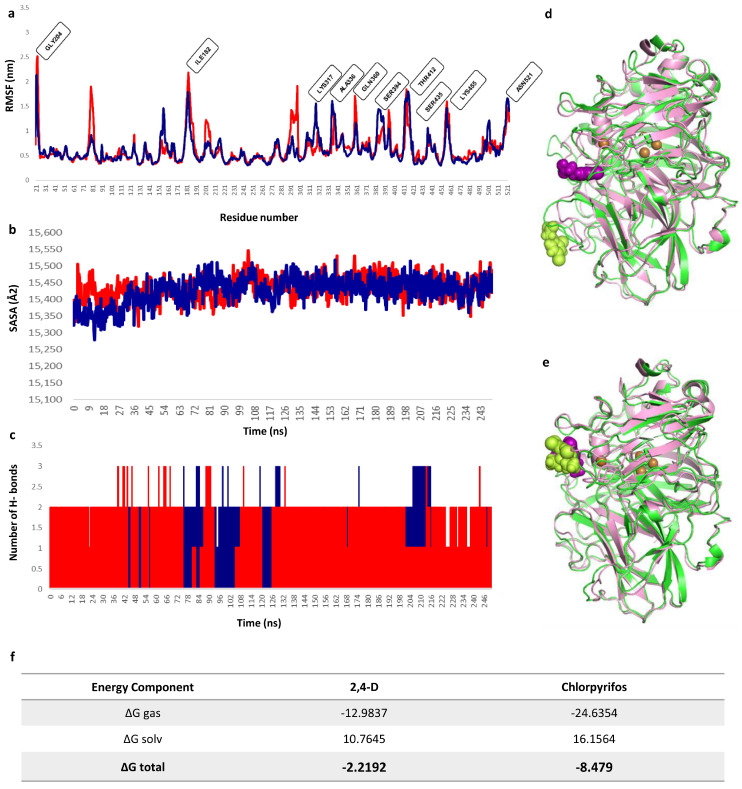
MDs simulation trajectory plots of *lacI* along with 2,4-dichlorophenoxyacetic acid (▬) and chlorpyrifos (▬) during 250 ns simulations. (**a**) The Cα root mean square fluctuation (RMSF) values of each amino acid residue for *lacI* in MDs simulations. (**b**) Solvent-accessible surface (SASA). (**c**) The number of hydrogen bonds formed during the protein–ligand interaction. (**d**,**e**) Structure representative of the behavior in the MDs simulation of the *lacI*-2,4D complex (**d**) and *lacI*–chlorpyrifos (**e**) at the beginning and at the end of 250 ns. The initial (pink structure) and final (green structure) conformations of the proteins are visualized, as well as the initial (purple structure) and final position (lime green structure) of the ligands. All imaging was performed in VMD. (**f**) Details of the results of MM/PBSA calculations on the complexes expressed in Kcal mol^−1^.

These results were supported by the root mean square fluctuation (RMSF) of the substrates; both compounds showed fluctuations around the same regions of the protein structure, highly consistent with the oscillations observed in the APO structure (Figure S3). In addition, an increase in mobility was recorded around loop 285–305, involved in the interaction with the 2,4-D compound in the final stretch of the MDs simulation. The RMSF of the active site amino acids along with their fluctuation can be observed in detail in Figure 9a.

To evaluate whether the binding of chlorpyrifos and 2,4-D caused conformational changes in the protein, the solute-accessible surface area (SASA) was calculated. Despite the high mobility observed at the binding site, especially for 2,4-D, structural changes were not significant. This suggests that the interactions of these compounds with the solvent were not significantly increased and are unlikely to cause conformational changes at the T2 and T3 sites (Figure 9b). Hydrogen bonding is crucial for substrate specificity, metabolism, and catalysis [64].

The results of molecular dynamics simulations with both substrates revealed that *lacl* formed several hydrogen bonds during the 250 ns simulation (Figure 9c). However, hydrophobic-type interactions prevailed, in agreement with the results of previous docking performed with both ligands and their ability to stabilize the benzene ring of the compounds. In particular, the His460 residue showed interactions for both compounds, at the onset of dynamics for 2,4-D and along the entire pathway for chlorpyrifos. This is remarkable because His460 is one of the histidines that coordinate the T1 site. On the other hand, 2,4-D prioritized interaction with D228 before moving out of the binding site, indicating a polar component within a predominantly apolar site. Taken together, these results suggest that both compounds interacted primarily throughout the entire active site, similar to the binding and docking results of ABTS in *T. versicolor* laccase.

#### MM/PBSA Calculation: Active Pose of Substrates

To understand the dynamic changes described above in terms of molecular structure, we examined the protein and substrate poses of the initial and final frames of the 250 ns MDs simulations (run 3), as shown in Figure 9d,e. The variation obtained in the arrangement of the substrates within the catalytic site is reflected in the differences in hydrogen bonding and hydrophobic interactions observed for each substrate at 50 ns and 250 ns (Table S5). To determine whether the dynamic stability of the active conformations leading to product formation is also related to the thermodynamic stability of the protein–substrate complexes, we assessed the binding affinity of MM/PBSA over the entire 250 ns of the MDs trajectories (Figure 9f), determining the molecular factors that influence the interaction with the different ligands evaluated.

**Figure 10:**
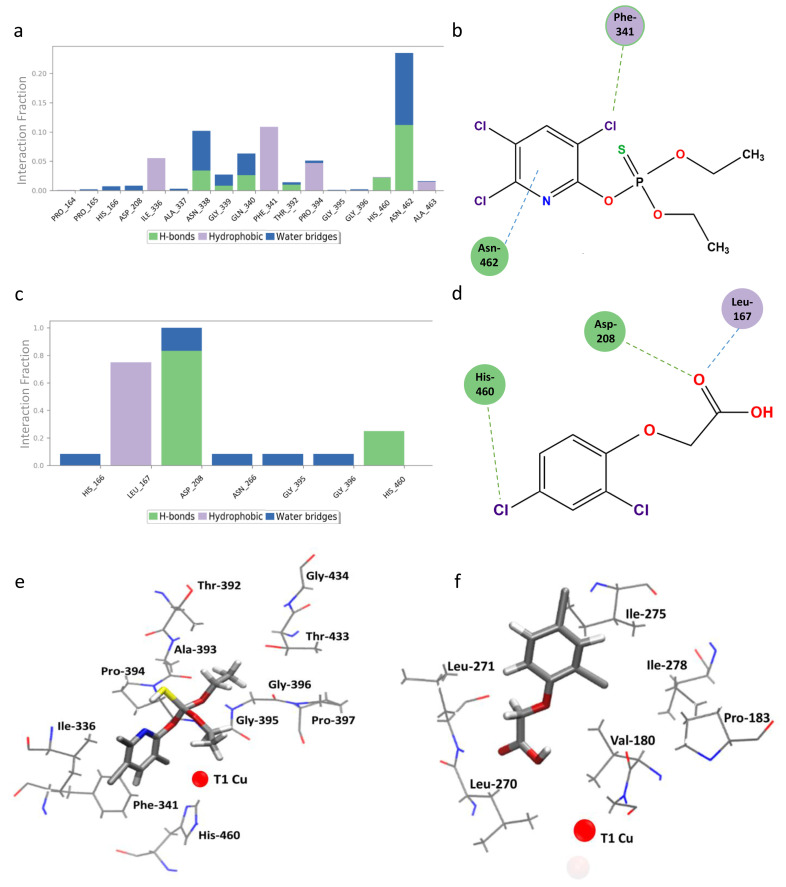
Protein–ligand interactions in *lacI*. (**a**) *lacI* residues interacting with chlorpyrifos. (**b**) Interactions with chlorpyrifos that were maintained for more than 10%. (**c**) *lacI* residues interacting with 2,4-D. (**d**) Interactions with 2,4-D that were maintained for more than 10%. Violet circles represent hydrophobic interactions, and green circles represent hydrogen bonds. (**e**) *lacI* residues interacting with chlorpyrifos in the representative MDs structure. (**f**) *lacI* residues interacting with 2,4-D in the representative MDs structure.

The analysis of the complexes revealed that the predominant interactions were hydrophobic, which agrees with the results of the previous coupling carried out with both ligands and the ability to stabilize the benzene ring of the compounds (Figure 10). In particular, residue His460 showed interactions for both compounds, at the beginning of the dynamics for 2,4-D and throughout the entire trajectory for chlorpyrifos. This is notable since His460 is one of the histidines that coordinate the T1 site. On the other hand, 2,4-D prioritized interaction with D228 before moving out of the binding site, indicating a polar component within a predominantly apolar site. Together, these results suggest that both compounds interacted mainly throughout the entire active site, similar to the binding of ABTS in *T. versicolor* laccase and the docking results with it.

## 3. Materials and Methods

### 3.1. Fungal Strains, Inoculum Preparation, and Chemicals

The WRF strain *P. brevispora* BAFC 633, previously isolated from dead logs in the Misiones rainforest, is deposited in the collection of filamentous fungal cultures of the Department of Biological Sciences, Faculty of Exact and Natural Sciences, University of Buenos Aires, Argentina. The strain was maintained through monthly subcultures in Petri dishes in malt extract of 12.7 g L^−1^ and 17 g L^−1^ agar (MEA, Biokar^®^ Diagnostics, Allonne, France) at 4 °C. The inoculum was obtained from active MEA plates incubated at 28 ± 1 °C for 7 days in the dark. The pesticides used for the biochemical studies, chlorpyrifos and 2,4-D, were obtained from Sigma-Aldrich^®^ (Ciudad Autónoma de Buenos Aires CABA, Argentina).

### 3.2. Laccase Tolerance and Secretion Assay in Solid Culture in the Presence of Pesticides

The tolerance of the fungal strain to pesticides was tested in Petri dishes (85 mm in diameter) containing 20 mL of MEA supplemented with different concentrations of pesticides: chlorpyrifos (0.1, 1, 1, 10, 100 mg L^−1^) and 2,4-D (1, 10, 100, 1000 mg L^−1^). Fragments of young mycelium, 5 mm in diameter and cut with a corkscrew from the edge of a 5-day-old *P. brevispora* BAFC 633 culture on MEA, were inoculated into the center of each Petri dish. Petri dishes inoculated with the fungus in the absence of pesticides were used as controls. Cultures were incubated in the dark at 28 ± 1 °C. Radial growth was measured daily until complete plate coverage was achieved. Fungal growth was modeled using a logistic equation [65]:

(1) $$D=\frac{D_{max}}{1+e^{\kappa \left(\tau -t\right)}}$$

where *D* is the fungal colony diameter, *D_max_* is the maximum diameter (set at 85 mm, corresponding to the diameter of the plates), *κ* is the fungal growth rate (m day^−1^), *τ* is the time required to reach half of *D_max_* (days), and t is the time (days). Fitting was performed using a least squares approach with nonlinear regression in InfoStat 2018p (Grupo InfoStat, Córdoba, Argentina) software [66].

Fungal growth τ was standardized as

Δτ = τP − τC,(2)

where τP is the value of the medium supplemented with the pesticide 2,4-D or chlorpyrifos and τC is the value of the control culture without pesticides to ignore the effect of the components of the culture medium.

A positive Δ*τ* value indicated the inhibition of fungal growth in response to pesticides and/or their toxic metabolites. In parallel, using the same experimental conditions, each plate was coated with 5 mM 2,6-dimethoxyphenol (DMP) in acetate buffer (pH 3.6) and incubated in the dark for 20 min to reveal laccase activity [31]. The appearance of the orange-yellow color in solid media, corresponding to the oxidation of DMP to 2,2′,6,6′-tetramethoxydibenzo-1,1′-diquinone [67], indicated a positive result [10].

### 3.3. Polyacrylamide Gel Electrophoresis

To establish the enzyme profile and molecular weight of laccase isoenzymes on pesticide-supplemented plates, the contents of the plates were frozen at −20 °C for 24 h. The plates were then thawed and centrifuged for 15 min at 10,000× *g*. The supernatant was used for electrophoretic separation through sodium dodecyl sulfate polyacrylamide gel electrophoresis (SDS-PAGE) (12% p v^−1^), followed by re-naturalization and detection with DMP [10,68]. The profile was compared with a molecular weight marker (Amersham, ECL RainbowMarker—Full Range, GE Healthcare, Chicago, IL, USA).

### 3.4. Effect of Pesticides on the Growth and Morphological Appearance of the Mycelium

Morphological changes due to pesticide addition were evaluated through direct macroscopic observation and scanning electron microscopy (SEM). For microscopic analysis, a sample of the mycelium from each treatment was collected and fixed with a solution containing formaldehyde, alcohol, and acetic acid (10:50:5). After dehydration with acetone, critical point drying with CO_2_, and gold plating, observations were made using a JEOL JSM 5800 LV scanning electron microscope (JEOL Ltd., Tokyo, Japan). To evaluate the effect of pesticides on mycelia, the diameter of fifty hyphae per treatment was measured using ImageJ 1.46r software.

### 3.5. Production of Laccase in Liquid Fermentation

*P. brevispora* BAFC 633 was grown in fermentation both in the presence and in the absence of CuSO_4_, a recognized inducer of laccase activity [33]. For this purpose, an agar plug (5 mm in diameter) of *P. brevispora* BAFC 633 grown on 5- to 7-day-old MEA plates was cut and transferred to 250 mL Erlenmeyer flasks in liquid medium (ME) containing 12.7 g L^−1^ of malt extract and 5 g L^−1^ of fermented corn liquor and CuSO_4_ (1 mM) with a final volume of 50 mL. Liquid cultures were incubated at 29 °C under steady-state conditions for 10 days. The liquid medium (supernatant) was then separated from the mycelium by filtration in a Büchner funnel using glass fiber filters (GF C^−1^) and frozen at −20 °C until use.

Laccase from *P. brevispora* BAFC 633 was concentrated as described in Fonseca et al. (2015) [10] and its molecular weight was verified through SDS-PAGE. The concentrated enzymatic fraction was used to perform enzymatic quantification and addition assays of chlorpyrifos and 2,4-D separately, at a final concentration of 10 mg L^−1^ for each pesticide; each experiment included a control without pesticides or CuSO_4_.

Laccase activity was assayed at 30 °C using 5 mM 2,6-dimethoxyphenol (DMP) as a substrate in a 0.1 M sodium acetate buffer (pH 3.6). The increase in the absorbance of the reaction mixture was monitored at 469 nm (ε469 = 27.5 mM cm^−1^) in a Shimadzu UV-3600 spectrophotometer (Shimadzu Corporation, Kyoto, Japan). Enzyme activity was expressed as International Units (U), defined as the amount of the enzyme needed to produce 1 µmol of product min^−1^ at 30 °C [31].

### 3.6. Effect of Pesticides on Laccase Activity

Chlorpyrifos and 2,4-D were added separately to the previously obtained enzymatic fractions at a final concentration of 10 mg L^−1^ for each one. pH measurements were performed at 30 °C in pH buffers ranging from 3.6 to 8, using DMP as the substrate. To determine pH stability, each treatment was incubated in different pH buffers ranging from 3.6 to 7, sampled at different intervals for 4 h at 30 °C, and then assayed for residual enzyme activity.

To define the optimal temperature, the enzymatic activity was measured at the optimal pH at different temperatures in a range of 20 to 90 °C. Thermal stability was investigated by measuring the residual activity after incubating the extract at different temperatures ranging from 20 to 70 °C by taking samples at intervals for 5 h. The residual activity was expressed as a percentage, considering the enzymatic activity at time zero as 100%.

### 3.7. Statistical Analysis

All assays were performed in triplicate. Data were processed using Microsoft Excel, and statistical analysis was performed using Statgraphics Centurion XVI.I software. The Anderson–Darling test was used to test for normality and Bartlett’s test was used to test for the homogeneity of variances. Data assuming a normal distribution were subjected to a one-way ANOVA followed by Tukey’s tests for analyses with more than two groups. Results were presented as the mean ± SD.

### 3.8. P. brevispora BAFC 633 Laccase Gene

The nucleotide (JQ728448.1) and amino acid (AFK30375.2) sequences of the laccase enzyme used in this study correspond to *lacI*, previously annotated, characterized, and reported in the GenBank database (https://www.ncbi.nlm.nih.gov/genbank (accessed on 3 May 2024)) [7].

### 3.9. Structure of the Ligands

The structures of 2,4-dichlorophenoxyacetic acid (CID: 16180) and chlorpyrifos (CID: 2730) were obtained from the Pubchem database Chemical Structure Search (https://pubchem.ncbi.nlm.nih.gov/ (accessed on 12 June 2024)).

### 3.10. Three-DimensionalModeling of Laccase

The model was built using Phyre 2.0 (Structural Bioinformatics Group, Imperial College London, United Kingdom) [69] and the crystallographic structure of *Trametes versicolor* laccase (PDBID: 1GYC) (http://doi.org/10.2210/pdb1gyc/pdb (accessed on 7 May 2024)) as a template. Geometry analysis was estimated using the server of the QMEAN Swiss Institute of Bioinformatics (SIB). To estimate the correct dihedral angle arrangement (ψ/Ф) in the model, a Ramachandran plot was generated by USFC Chimera 1.10.227 (https://www.cgl.ucsf.edu/chimera/ (accessed on 7 May 2024) [70]. SuperPose 1.0 (http://superpose.wishartlab.com/ (accessed on 9 May 2024) was used to determine the structural divergence.

The MolProbity server (http://molprobity.biochem.duke.edu/ (accessed on 7 May 2024)) was used to evaluate and validate the crystallographic resolution. The architecture was analyzed using the VADAR 1.8 program (http://vadar.wishartlab.com/ (accessed on 10 May 2024)) [41].

### 3.11. Characterization and Prediction of Interaction Sites in Catalytic Pockets

DoGSiteScorer (http://dogsite.zbh.uni-hamburg.de/ (accessed on 13 June 2024)) was used to identify potential pockets in the models and assess the probability of binding a compound [71]. The ideal_sites.py module available in the MGLTools 1.5.7 package performed the calculation of ideal interactions [72]. The sites found were compared with the crystallographic structure of *Bacillus subtilis* laccase with co-crystallized ABTS (2,2′-azino-bis (3-ethylbenzothiazoline-6-sulfonic acid)) (PDBID: 3ZDW) (http://doi.org/10.2210/pdb3zdw/pdb (accessed on 21 June 2024)).

### 3.12. Evaluation of Molecular Docking

The AutoDock 4.2.6 (Olson Lab, La Jolla CA, USA) program was used for the docking calculations. The position for the grid center was estimated from the center of mass of the ABTS ligand in the reference complex (PDBID: 3ZDW), which is close to the T1 site. Energy maps were calculated for each atom type of each ligand. The grid covered in all cases the totality of the amino acids present in the active site with a resolution of 0.375 Å and a size of 30 × 32 × 34 Å. For each docking experiment, 100 different runs were performed, and the results were grouped according to a cutoff of 2 Å. The bias docking method was used for pharmacophore sites [73], calculated using the ideal_site.py module of AutoDockTools 1.5.7. Compounds and receptors were prepared using the prepare_ligand4.py and prepare_receptor4.py modules, respectively. The analysis of docking results was carried out using Discovery Studio v19.1.0.18287 (Biovia, San Diego, CA, USA) [74] and Visual Molecular Dynamics (VMD 1.8.5 University of Illinois, Urbana, IL, USA) [75].

### 3.13. Molecular Dynamics Simulation (MDS)

All MDSs were performed using NAMD 3.0.0 (University of Illinois, Urbana, IL, USA) software [76]. The force field used for the simulations was Charmm27 (CHARMM Development Project, Cambridge, MA, USA). Receptors were constructed using NAMD’s System Builder, solvated in a TIP3P octahedral mixed solvent box with 0.15 M Na^+^ and Cl ions^−^. The prepared systems were then energy-minimized and equilibrated for 10 ns. Ligand parameters and topologies were calculated using the Charmm27 force field with the Ligand Reader and Modeler online software (http://www.charmm-gui.org/?doc=input/ligandrm (accessed on 23 July 2024)) [77]. The generated parameters and topology files were then loaded into VMD to read the protein–ligand complexes easily and without errors and then perform the simulation step. Tcl harmonic forces were applied to hold the Cu^2+^ in place. To analyze the thermodynamic stability of the enzyme and receptor–ligand complexes, the root mean square deviation (RMSD), root mean square fluctuation (RMSF), hydrogen bonds (H-BOND), and solute-accessible surface area (SASA) were calculated. To qualitatively analyze the binding of 2,4-D and chlorpyrifos to the *lacI* receptor, three simulations were performed, two standard ones of 50 ns each (runs 1 and 2) and a longer one of 250 ns (run 3), in all cases using random seeds and velocities in order to guarantee the independence of the simulations [78]. The binding stability and free energy in each complex were determined during the longer MDS of 250 ns.

#### Binding Free Energy Calculations

For all simulations corresponding to the complexes, binding enthalpy contributions were calculated using the MM/PBSA (Molecular Mechanics Poisson–Boltzmann Surface Area) method. The MMPBSA.py module of the AMBER18 package was used [79]. A total of 100 frames were processed from the trajectories, and the system’s net energy was estimated using the following equation:

ΔG Binding = ΔG Complex − ΔG Receptor − ΔG pesticide (3)

Each of the above-mentioned terms requires the calculation of multiple energy components, including van der Waals energy, electrostatic energy, internal energy from molecular mechanics, and polar contribution to solvation energy.

## 4. Conclusions

This study demonstrated that *P. brevispora* BAFC 633 was able to grow in the presence of 2,4-D and chlorpyrifos, showing notable morphological and ultrastructural changes in the mycelium, such as reduced hyphal and basidiospore diameters, along with the secretion of two laccase isoenzymes. Laccase enzymatic activity increased significantly in the presence of both pesticides, with greater activity observed with chlorpyrifos compared to 2,4-D. Computational studies supported this finding by revealing differential ligand behavior in the T1 site of laccase. Molecular docking, MM/PBSA, and molecular dynamics analyses showed higher affinity energy with chlorpyrifos, resulting in greater ligand stability within the active site during prolonged simulations, in contrast to the higher mobility detected for 2,4-D.

However, although these results suggest significant potential for bioremediation, important challenges remain regarding practical applicability. Enzymatic stability, process scalability, and environmental interactions are critical factors that must be addressed for the effective implementation of these strategies in field conditions, where additional variables such as the presence of other contaminants, pH, and ambient temperature fluctuations must be considered.

Complementary computational analysis has also provided an in-depth understanding of the molecular mechanisms involved in the interaction between laccase and pesticides, revealing differential affinities and the residues involved in various static and dynamic scenarios. These results lay a solid foundation for future studies that delve into interactions at the atomic level using tools such as X-ray crystallography or nuclear magnetic resonance. Additionally, it will be crucial to investigate whether *P. brevispora* BAFC 633 laccase can not only interact with but also effectively degrade compounds like chlorpyrifos, which could lead to targeted enzyme modifications to optimize its specificity and catalytic efficiency in biotechnological and environmental applications.

## Figures and Tables

**Table 1:** *P. brevispora* BAFC 633 τ (time to reach half of maximum diameter) (days) and growth rate *k* (growth diameter/time) on solid media in the presence (with) and absence (without) of 2,4-D and chlorpyrifos (CP). Data are means ± standard deviations. Means with different letters are significantly different (*p* < 0.05).

Pesticide	Concentration [mg L^−1^]	*k*	τ
without	N/D	1.30 ± 0.02 ^a^	2.13 ± 0.03 ^c^
with 2.4-D	1	1.21 ± 0.08 ^ab^	2.22 ± 0.02 ^bc^
	10	1.05 ± 0.02 ^cd^	2.4 ± 0.01 ^b^
	100	0.58 ± 0.10 ^f^	3.54 ± 0.30 ^a^
	1000	-	-
with CP	0.1	1.15 ± 0.06 ^bc^	2.16 ± 0.07 ^c^
	1	1.01 ± 0.05 ^de^	2.23 ± 0.06 ^bc^
	10	0.93 ± 0.03 ^e^	2.35 ± 0.04 ^bc^
	100	0.66 ± 0.10 ^f^	3.55 ± 0.27 ^a^

**Table 2:** Amino acid residues involved in the interaction between ligands and the laccase model *lacI*.

Ligand	2,4-D	Chlorpyrifos
residue AA	Gly395	His166
	Pro397	Leu167
	Asn266	Asp208
	Ala267	Gly395
	Ile457	Ile457
	Pro209	Phe459
	Ser210	His460
	Asp208	Pro394
	Gln268	Ala267
	Pro165 *	Pro165
	His166	Ala463
	Leu167 *	Phe341

* denotes residues that participate in the hydrogen bonding interaction during docking.

**Table 3:** Ligand RMSD analysis during the four MDs runs.

Analysis of the Ligand Movement from the T1 Site				
Complex	50 ns	100 ns	150 ns	250 ns
*lacI*-2,4D	The ligand remains at the active site.	Moves away very soon, to a lower distal position, at ~80 ns.	Moves very fast, remains distally moving in and out for ~150 ns.	It remains attached at the distal site.
*lacI*-chlorpyrifos	The ligand remains in the active site, fluctuating in its position.			

## Data Availability

The data supporting this study’s findings are available from the corresponding author upon reasonable request.

## Supplementary Materials

The following supporting information can be downloaded at: https://www.mdpi.com/article/10.3390/ijms252312527/s1.
