# Supplementary material for: In Vitro and Computational Response of Differential Catalysis by Phlebia brevispora BAFC 633 Laccase in Interaction with 2,4-D and Chlorpyrifos
Source: Int J Mol Sci. 2024 Nov 22;25(23):12527. doi: 10.3390/ijms252312527 (PMC11641778; doi:10.3390/ijms252312527)
Supplement: Supplementary file 1 [file ijms-25-12527-s001.zip › ijms-3306554-supplementary tables.pdf]

Table S1. Δτ in solid media for *Phlebia brevispora* BAFC 633 grown in the presence of 2,4-D and Chlorpyrifos at different concentrations. Data are mean ± standard deviation.

Treatment	Δ*t*
2,4D [1 mg/L]	0.09 ± 0.01
2,4D [10 mg/L]	0.27 ± 0.02
2,4D [100 mg/L]	1.41 ± 1.02
2,4D [1000 mg/L]	NG *
CP [0.1 mg/L]	0.03 ± 0.04
CP [1 mg/L]	0.10 ± 0.03
CP [10 mg/L]	0.22 ± 0.01
CP [100 mg/L]	1.42 ± 0.24

*NG. not grow

Table S2. Half-life of the laccase activity of *P. brevispora BAFC 633* in supernatants of cultures incubated at different temperatures (at optimal pH) and pHs (at optimal temperature)

Evaluated characteristic	Without $CuSO_4$		With $CuSO_4$	
	With 2,4 D	With CP	With 2,4 D	With CP
Half- life al pH 3.6	4 h	>4 h	> 4 h	> 4 h
Half- life al pH 4.8	> 4 h	>4 h	> 4 h	> 4 h
Half- life al pH 5	> 4 h	> 4 h	> 4 h	> 4 h
Half- life al pH 6	> 4 h	> 4 h	> 4 h	> 4 h
Half- life al pH 7	> 4 h	> 4 h	> 4 h	> 4 h
Half- life at 20 °C	2,5 h	4,5 h	> 5 h	> 5 h
Half- life at 30 °C	> 5 h	3,7 h	> 5 h	> 5 h
Half- life at 40 °C	> 5 h	3,5 h	> 5 h	5 h
Half- life at 50 °C	1,5 h	2,2 h	4,5 h	3,1 h
Half- life at 60 °C	3 h	2,5 h	1,3 h	2,2 h
Half- life at 70 °C	3,5 h	2,2 h	3 h	2,8 h

Note: The half-life of laccase activity is expressed as the time necessary for the enzymatic activity to be reduced by 50 %

Table S3. Formation of angles between amino acid atoms and copper ions in template (1GYC) and generated (lacI) model

**Active site**	***T. versicolor***		**lacI**	
	**Angle formation between amino acid and copper ion**	**Angle (°)**	**Angle formation between amino acid and copper ion**	**Angle (°)**

**T1**	His458 (ND1)→Cu1→Cys453 (SG)	129.000	His480 (ND1)→Cu1→Cys475 (SG)	132.86
	His395 (ND1)→Cu1→Cys453 (SG)	126.160	His418 (ND1)→Cu1→Cys475 (SG)	125.97
	His458 (ND1)→Cu1→His395 (ND1)	104.438	His480 (ND1)→Cu1→His418 (ND1)	98.85
**T2**	His398 (NE2)→Cu4→His64 (NE2)	174.324	His421 (NE2)→Cu4→His87 (NE2)	173.26
**T3**	His111 (NE2)→Cu2→His400 (NE2)	101.998	His134 (NE2)→Cu2→His423 (NE2)	90.65
	His452 (NE2)→Cu2→His400 (NE2)	101.268	His474 (NE2)→Cu2→His423 (NE2)	100.92
	His111 (NE2)→Cu2→His452 (NE2)	106.002	His134 (NE2)→Cu2→His474 (NE2)	108.72
	His454 (NE2)→Cu3→His66 (NE2)	107.135	His476 (NE2)→Cu3→His89 (NE2)	106.55
	His109 (NE2)→Cu3→His66 (NE2)	125.396	His132 (NE2)→Cu3→His89 (NE2)	127.00
	His454 (NE2)→Cu3→His109 (NE2)	111.511	His476 (NE2)→Cu3→His132 (NE2)	112.65

Table S4. VADAR prediction model molecular characteristics.

Characteristic	lacI	Expected value (EV)
α-helix	25 (5%)	-
β-sheet	244 (49%)	-
Coil	222 (45%)	-
Turn	144 (29%)	-
Hydrogen bond average distance (Å)	2.1 sd=0.4	2.2 sd=0.4
Hydrogen bond average energy (kJ mol-1)	-2.0 sd=0.9	-2.0 sd=0.8
Number of residues with hydrogen bonds	328 (66%)	368 (75%)
Helix average (Φ)	-63.9 sd=7.9	-65.3 sd=11.9
Helix average (ψ)	-32.7 sd=30.4	-39.4 sd=25.5
ω average angle (>90°)	178.2 sd=7.5	180.0 sd=5.8
Number of residues with ω angles <90°	6 (1%)	-

Total ASA (Å2)	17926.9	17569.7
Molecular weight (kDa)	53147.10	-

Table S5. Non-covalent interactions of selected compounds with lacI obtained via MD simulation at 50 ns and 250 ns.

Ligands	Hydrogen bonds 50 ns	250 ns	Hydrophobic interactions 50 ns	250 ns
2,4-Dichlorophenoxyacetic acid		LEU271(3.67)	PHE241,PRO397,ASN266, SER210, THR433	ILE275, VAL180, LEU270, PRO183, GLY181
Chlorpyrifos	HIS460 (4.85)	GLY395 (3.26)	HIS166, ASP208, PRO394, GLY395, ILE457	PRO394, GLY396, THR433, ILE336, PHE341.
