# Supplementary figures and images for: In Vitro and Computational Response of Differential Catalysis by Phlebia brevispora BAFC 633 Laccase in Interaction with 2,4-D and Chlorpyrifos
Source: Int J Mol Sci. 2024 Nov 22;25(23):12527. doi: 10.3390/ijms252312527 (PMC11641778; doi:10.3390/ijms252312527)

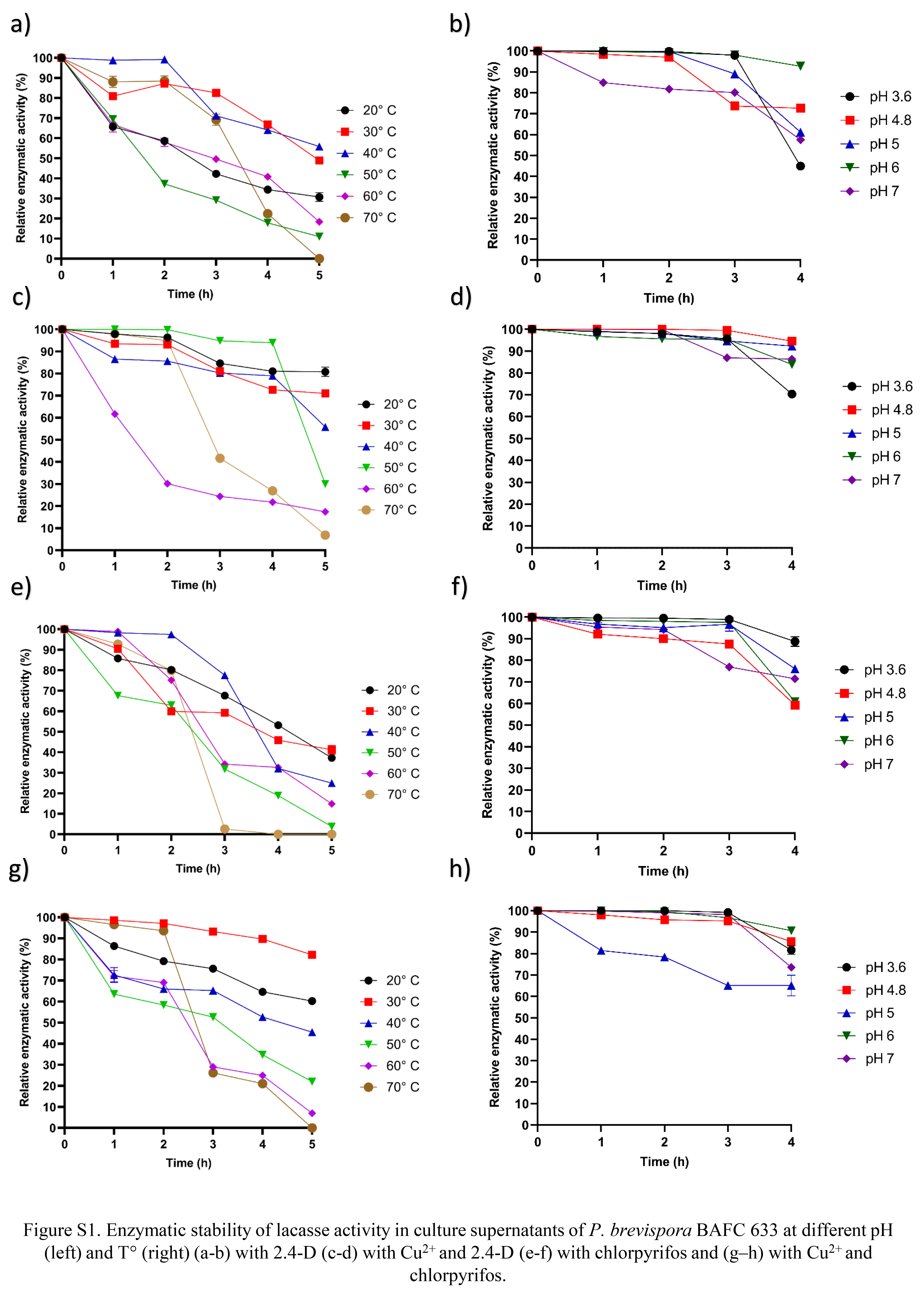

Supplement: Supplementary file 1 [file ijms-25-12527-s001.zip › Figure S1.tif]

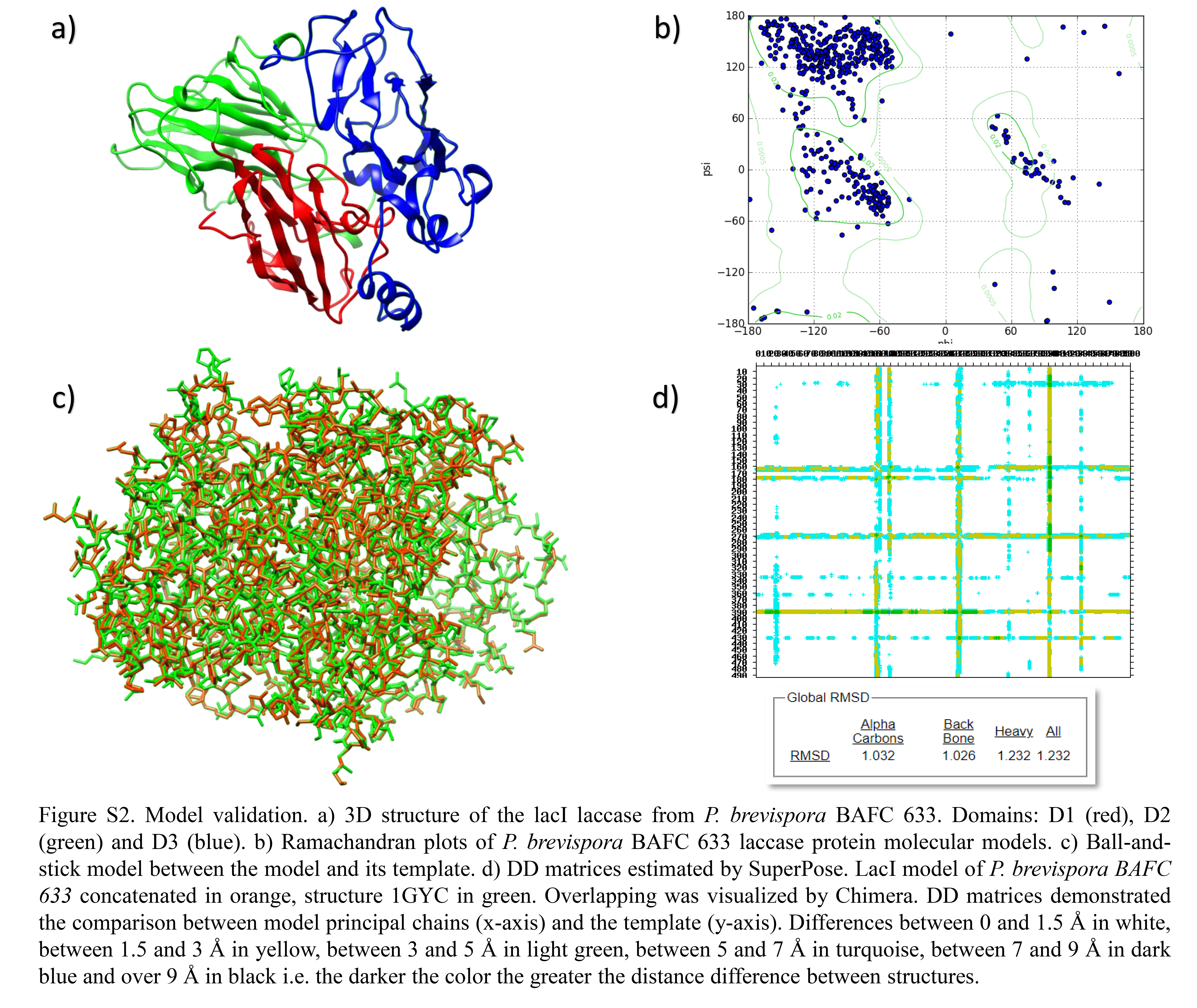

Supplement: Supplementary file 1 [file ijms-25-12527-s001.zip › Figure S2.tif]

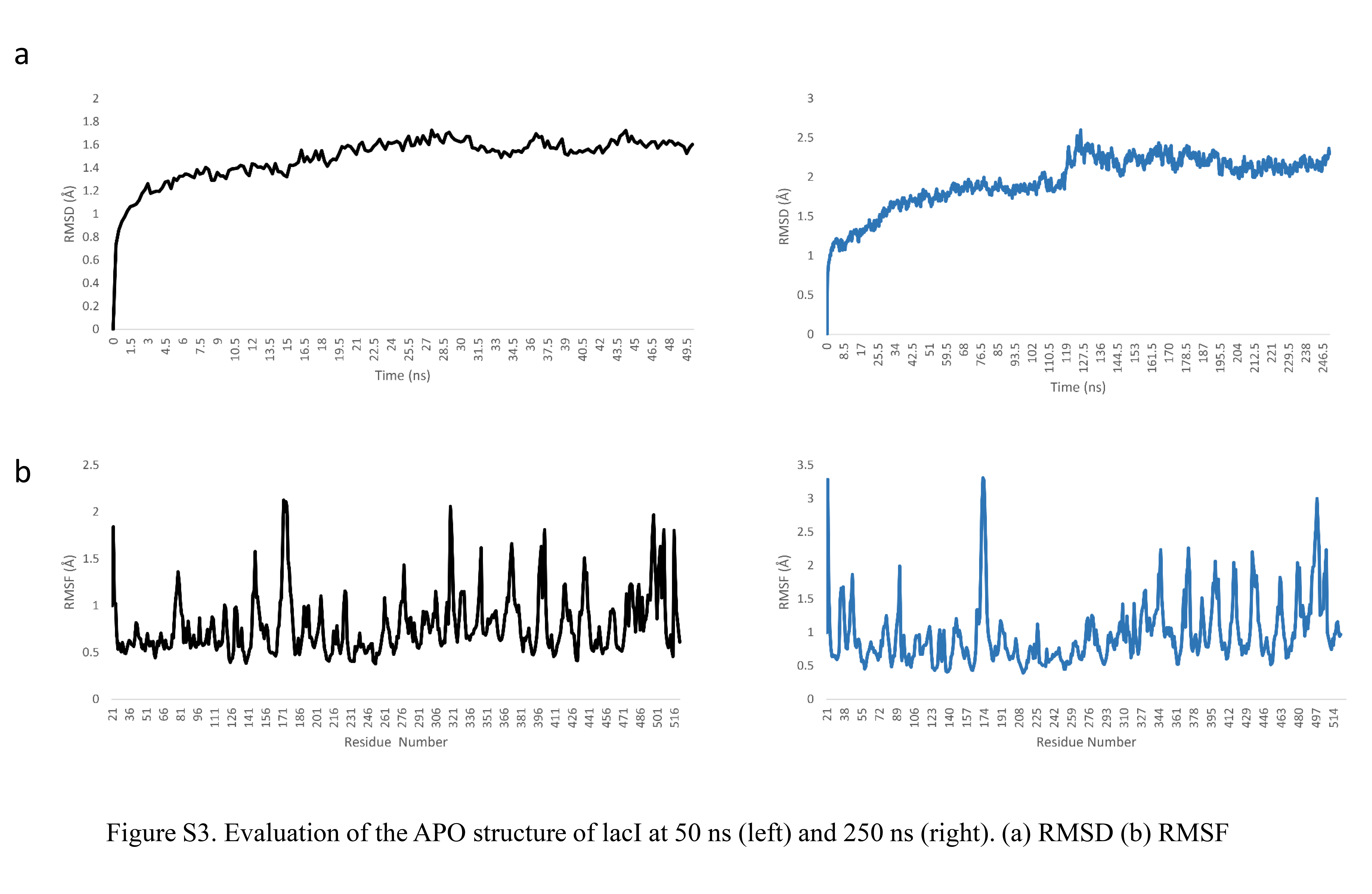

Supplement: Supplementary file 1 [file ijms-25-12527-s001.zip › Figure S3.tif]
